# Using RNA-seq to determine the transcriptional landscape and the hypoxic response of the pathogenic yeast *Candida parapsilosis*

**DOI:** 10.1186/1471-2164-12-628

**Published:** 2011-12-22

**Authors:** Alessandro Guida, Claudia Lindstädt, Sarah L Maguire, Chen Ding, Desmond G Higgins, Nicola J Corton, Matthew Berriman, Geraldine Butler

**Affiliations:** 1 School of Medicine and Medical Science, Conway Institute, University College Dublin, Belfield, Dublin 4, Ireland; 2 School of Biomolecular and Biomedical Science, Conway Institute, University College Dublin, Belfield, Dublin 4, Ireland; 3 Department of Pharmacology and Cancer Biology, Duke University Medical Center, Box 3813 Research Drive, Durham, North Carolina, USA; 4 Pathogen Genomics Group, Wellcome Trust Sanger Institute, Cambridge, UK

**Keywords:** Transcriptional profiling, pathogenesis, RNA-seq, Candida

## Abstract

**Background:**

*Candida parapsilosis *is one of the most common causes of *Candida *infection worldwide. However, the genome sequence annotation was made without experimental validation and little is known about the transcriptional landscape. The transcriptional response of *C. parapsilosis *to hypoxic (low oxygen) conditions, such as those encountered in the host, is also relatively unexplored.

**Results:**

We used next generation sequencing (RNA-seq) to determine the transcriptional profile of *C. parapsilosis *growing in several conditions including different media, temperatures and oxygen concentrations. We identified 395 novel protein-coding sequences that had not previously been annotated. We removed > 300 unsupported gene models, and corrected approximately 900. We mapped the 5' and 3' UTR for thousands of genes. We also identified 422 introns, including two introns in the 3' UTR of one gene. This is the first report of 3' UTR introns in the Saccharomycotina. Comparing the introns in coding sequences with other species shows that small numbers have been gained and lost throughout evolution. Our analysis also identified a number of novel transcriptional active regions (nTARs). We used both RNA-seq and microarray analysis to determine the transcriptional profile of cells grown in normoxic and hypoxic conditions in rich media, and we showed that there was a high correlation between the approaches. We also generated a knockout of the *UPC2 *transcriptional regulator, and we found that similar to *C. albicans*, Upc2 is required for conferring resistance to azole drugs, and for regulation of expression of the ergosterol pathway in hypoxia.

**Conclusion:**

We provide the first detailed annotation of the *C. parapsilosis *genome, based on gene predictions and transcriptional analysis. We identified a number of novel ORFs and other transcribed regions, and detected transcripts from approximately 90% of the annotated protein coding genes. We found that the transcription factor Upc2 role has a conserved role as a major regulator of the hypoxic response in *C. parapsilosis *and *C. albicans*.

## Background

*Candida *species are the causative agents of 8-10% of hospital-acquired bloodstream infections worldwide [1]. *Candida albicans *remains the most common, but other species (such as *Candida tropicalis*, *Candida parapsilosis *and *Candida glabrata*) are increasing in frequency. *C. parapsilosis *is currently the second most commonly isolated species in Latin America, and it is particularly prevalent in children less than 1 year old [2]. *C. parapsilosis *is also a frequent cause of infection in Europe [2]. Pathogenesis of *C. parapsilosis *is associated with use of indwelling medical devices and with high glucose feeds [3]. This species is often found on the hands of health care workers, and has been responsible for several outbreak infections [4-9].

Until recently, *C. parapsilosis *isolates were believed to be highly heterogeneous, and were divided into Groups I, II and III [10,11]. Group I isolates are now defined as *C. parapsilosis*, whereas Groups II and III have been redesignated as the closely related species *C. orthopsilosis *and *C. metapsilosis *[12]. *C. parapsilosis *Group I isolates are very homogeneous, and are very difficult to distinguish using standard molecular methods such as RAPD profiling [13,14]. Sequencing the *C. parapsilosis *genome revealed that there are very few differences between the diploid chromosomes, with only one single nucleotide polymorphism (SNP) per 15,553 bases [15]. This may indicate that the species has undergone a recent population bottleneck, perhaps related to the lack of a sexual cycle, and the loss of one mating type [15-17].

The *C. parapsilosis *genome was sequenced in 2009, together with the genomes of 5 other *Candida *species [15]. There are currently two independent annotations of the genome. One was generated from an early assembly (consisting of 143 contigs) using an automated annotation pipeline and contains 5733 gene models, which are identified with the prefix "cpag" ([15]http://www.broad.mit.edu/annotation/genome/candida_group/MultiHome.html). A second annotation produced by our laboratory using the same assembly characterized 5,834 gene models (including 12 mitochondrial genes), which are identified with the prefix "cpar" [18,19]. The initial annotations proved useful for large-scale comparative analysis [15,19] and for the design and application of transcriptional profiling [18,20]. However, neither the "cpag" nor "cpar" annotations included predictions of introns in any genes. Moreover, the genome sequence was also subsequently improved so that most of the current assembly consists of 8 contigs, corresponding to complete chromosomes [15]. We have now used this assembly as the basis for a new annotation of the *C. parapsilosis *genome.

We applied next-generation sequencing to explore and better define the transcriptional landscape. More than 330 million Illumina reads were generated from seven different growth conditions (including varying temperatures, media and oxygen levels). Strand-specific and long read libraries were included in the experimental design. We used these data to refine gene models, determine intron boundaries, identify nTARs and eliminate overlapping gene models lacking transcriptional support. We also used both RNA-seq and microarray analysis to determine the transcriptional response of *C. parapsilosis *to growth on rich media in hypoxic conditions, and we found a high correlation between the two approaches. Finally, we used transcriptional profiling to investigate the role of the Upc2 transcription factor as a regulator of the hypoxic response.

## Results and discussion

### Determining the transcriptional profile of *Candida parapsilosis*

Next-generation sequencing (Illumina) was used to determine the transcriptome of *C. parapsilosis*, and to annotate the genome. In order to maximize coverage, we generated libraries from several conditions, including varied temperature (30°C, 37°C), media (YPD, YPglycerol, BMW, SD) and oxygen levels (21% and 1% O_2_) (Additional file 1). BMW media is designed to minimize growth differences between different species [21]. Strand-specific libraries were prepared from some samples grown in normoxia and hypoxia, and for a wildtype strain and a strain carrying a knockout of an ortholog of the *Candida albicans UPC2 *transcription factor, a major regulator of ergosterol synthesis and the hypoxic response [22-24]. Most reads were approximately 36 bases long; two long read (78 base) libraries were also generated commercially, from cells grown in YPD at 30°C. Approximately 280 million reads (including 26 million reads from the rDNA locus) were mapped to single locations in the genome using TopHat [25].

**Figure 1:**
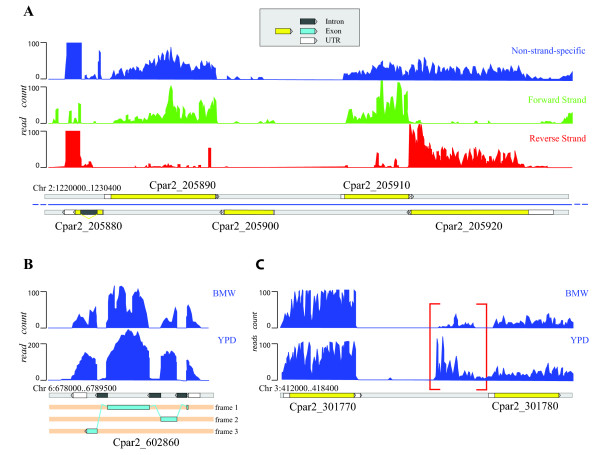
**Annotation of the *C. parapsilosis *genome**. (A) Mapping of reads to a region of chromosome 2 (Contig005809). In the top panel, the transcribed strand is not identified. Strand-specific libraries shown below easily distinguish between the adjacent genes Cpar2_205910 and Cpar2_205920. Cpar2_205880 is expressed at a higher level than the other genes in the region. It also contains a short intron. (Data is from YPD media at 30°C for both strand-specific and non-specific information, from different experiments). (B) Gene with multiple introns (Cpar2_602860). This is an ortholog of *orf19.4193 *in *C. albicans*, which also has 3 introns at equivalent positions. Intronic regions in *C. parapsilosis *are well defined by an abrupt drop in the coverage abundance. (C) Identification of a novel transcriptional active region (nTAR). Transcribed regions not associated with annotated features were designated as nTARs. One potential example, which is a homolog of the U14 small nucleolar RNA from *S. cerevisiae *(snR128), is indicated with a bracket.

We produced a new annotation (Cpar2 prefix) by combining the RNA-seq data with the existing (cpag and cpar) annotations of the *C. parapsilosis *genome [15,18] and visualizing using the Artemis browser [26] (Figure 1). We identified 395 novel gene models (including 120 with introns) that were not included in the cpag annotation described in Butler *et al *[15,18] (Table 1, Table 2). We removed 318 overlapping gene models, and we corrected 664 by altering the position of start and stop codons, removing in-frame stop codons or identifying internal introns (Table 2). We also identified 26 pseudogenes that are well conserved with other *Candida *species, but the reading frames are interrupted by stop codons. In total, the Cpar2 annotation includes 5810 protein-coding genes and 91 potential tRNA genes that were predicted using tRNAscan-SE [27] (Table 1). We detected expression (FPKM, fragments per kilobase of transcript per million fragments mapped, value higher than 10) of 94% of the total protein-coding genes in the Cpar2 annotation.

**Table 1:** Details of the Cpar2 annotation of the *C. parapsilosis *genome

Protein coding genes	
Single-exon gene models	5423
Multi-exon gene models:	
Genes with introns in CDS only^**1**^	353
Genes with introns in UTR	34
Total protein coding genes	5810
Pseudogenes	26
Total protein-coding genes and pseudogenes	5836
**tRNA genes**	91

**rRNA genes**	4

**nTARs**	95

**Genes with mapped 5' UTRs**	4682
Genes with overlapping 5' UTRs	834

**Genes with mapped 3' UTRs**	3532
Genes with overlapping 3' UTRs	2087

^**1**^Two genes have introns in both 5'UTR and CDS sequence.

**Table 2:** Comparison of protein-coding predictions in the cpar and cpag annotations to the Cpar2 annotation

	cpar^1^	cpag^2^
**Number of gene models in original set** (all single-exon)	5822	5733

**Unmodified in Cpar2**	5195	4486

**Modified in Cpar2^3^**	442	929

**Removed in Cpar2**	185	318

**Additional gene models in Cpar2^3^**	173	395

^1^Annotation from Rossignol *et al *[18]

^2^Annotation from Butler *et al *[15]

^3^New and modified ORFs include the identification of 422 introns in 387 genes, listed in Additional file 4.

To identify the boundaries of the 5' and 3' untranslated regions (UTRs) we looked for continuous read coverage (> 2 reads) that extended beyond the open reading frames. It was not always possible to correctly establish the UTR boundary, particularly where intergenic regions are small. Where possible, strand specific data was used to differentiate the UTRs of genes that are on opposite strands. We identified 5' UTRs for 4682 gene models, plus an additional 834 gene models where the predicted UTR overlaps with a neighboring annotated feature (Additional file 2). For 295 5' UTRs and 195 3' UTRs, the expression level was not high enough to analyze. Genes with long 5' UTRs (> 500 bp; 295 gene models) are enriched for regulatory GO processes (Additional file 3) suggesting that there is a correlation between long UTR regions and regulatory function. This has also been reported in *C. albicans *[28]. We identified 3532 3' UTRs, of which 135 are longer than 500 bp. These are not enriched for any GO term. We were unable to define the 3' UTR for 2080 gene models because they overlapped with the UTR or coding region of the neighboring gene.

### Intron Discovery

One of the main advantages of RNA-seq is that it can be used to characterize introns. TopHat [25] identifies reads that overlap a splice junction, and aligns them to either side of a canonical splice site. We manually curated each candidate predicted by TopHat by examining the overall read coverage, verifying the presence of canonical (GT/AG) splice sites, and comparing to orthologous genes from closely related species. We found RNA-seq evidence supporting 368 novel introns, many in open reading frames not described in the cpag and cpar annotations. Most of the introns (70%) were identified from 13.8 million reads of 78 base pairs (Additional file 1); the longer reads were considerably more useful than the shorter ones. To identify introns in genes that are not expressed in the conditions we used, we compared the *C. parapsilosis *genome to gene models predicted in *C. albicans *[28-31]. We identified an additional 55 introns that are conserved between *C. albicans *and *C. parapsilosis*.

**Figure 2:**
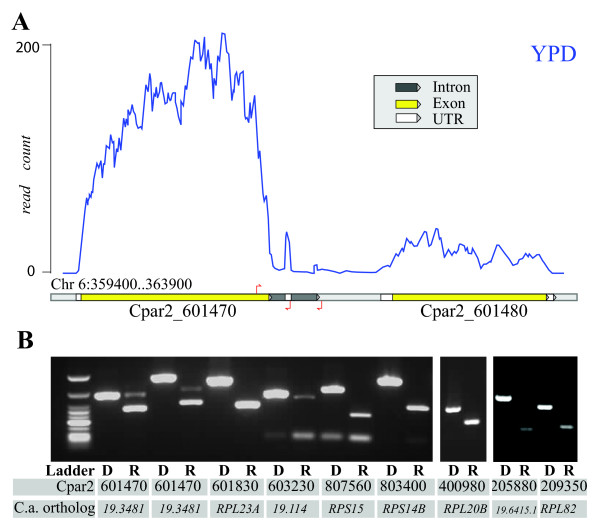
**Identification of introns**. (A) Non strand-specific RNA-seq (data is from YPD media at 30°C) showing the expression level for gene Cpar2_601470. Although expression of the 3' UTR region is low, two potential introns were identified. The red arrows show the location of oligonucleotide primers designed to confirm the presence of the introns. (B) Intron validation by PCR. Pairs of oligonucleotide primers flanking the intronic region were used to amplify products from genomic DNA (D) or cDNA (R). Introns were confirmed in the 3' UTR of Cpar2_601470, the 5' UTRs of Cpar2_400980 and Cpar2_205880, and within the coding sequence of 5 other genes (details of expected sizes are shown in Additional file 8). The small bands in Cpar2_601830 and Cpar2_807560 are likely due to the presence of excess primers or to nonspecific amplification.

Our final annotation includes a total of 422 introns in 387 genes (Additional file 4). Whereas most lie within coding sequences, 34 introns are located in 5' UTRs, and there are two in 3' UTRs. We selected a sample of eight gene models for validation by RT-PCR: one with two 3'UTR introns, two with introns in the 5' UTR, and five with introns within the coding region (Figure 2). The selected genes included orthologs of four that are known to contain introns in *C. albicans*, which are conserved in *C. parapsilosis *(Figure 2, Additional file 5). The *C. parapsilosis *orthologs of *RPL20 *(Cpar2_400980) and *RPS29A *(Cpar2_205880) contain introns in the 5'UTR regions that are spliced during growth on YPD (Figure 2). These are not annotated in *C. albicans*. In addition, *orf19.114 *has no annotated intron in *C. albicans*, but its *C. parapsilosis *ortholog (Cpar2_603230) contains an intron in the coding sequence. It is possible that many of these differences are due to errors in annotation in *C. albicans*.

The two 3' UTR introns we identified by RNA-seq are both in the same gene, Cpar2_601470. We confirmed both by RT-PCR (Figure 2). Cpar2_601470 is an ortholog of *C. albicans orf19.3481 *(gene of unknown function), which has no known introns. Although 3' UTR introns have been reported in animals and plants [32] and in fungi such as *Cryptococcus *[33], no 3' UTR introns have been identified previously in *Candida *or *Saccharomyces *species. Introns in 3' UTRs are assumed to be rare because structures after stop codons are likely to subject the mRNA to nonsense-mediated decay [33]. The 3' exons in Cpar2_601470 are transcribed at a much lower level than the reading frame (Figure 2A), and unspliced as well as spliced products are present (Figure 2B). It is therefore possible that the 3' UTR introns are important for the regulation of expression of this gene.

The total numbers of introns are similar in *C. albicans *(431 introns in 387 genes [34]) and *C. parapsilosis *(422 introns in 387 genes). The 5' and 3' splice site consensus are also very similar (Additional file 6). However, the median length is smaller in *C. parapsilosis *(68 bp) than in *C. albicans *(84 bp) (Additional file 6). For many other *Candida *species, the genome annotations are not accurate enough to compare intron locations. Any comparative analysis is therefore restricted to genomes were introns have been predicted, or experimentally identified. For example, in *C. albicans *introns have been carefully validated using both experimental and predictive methods [28-31]. The genome annotations of *C. dubliniensis *and *Debaryomyces hansenii *also include predictions of a significant numbers of introns [35,36]. We therefore compared the intronic landscape of these three species with *C. parapsilosis*. Orthologous genes were identified using the Candida Gene Order Browser [19].

**Figure 3:**
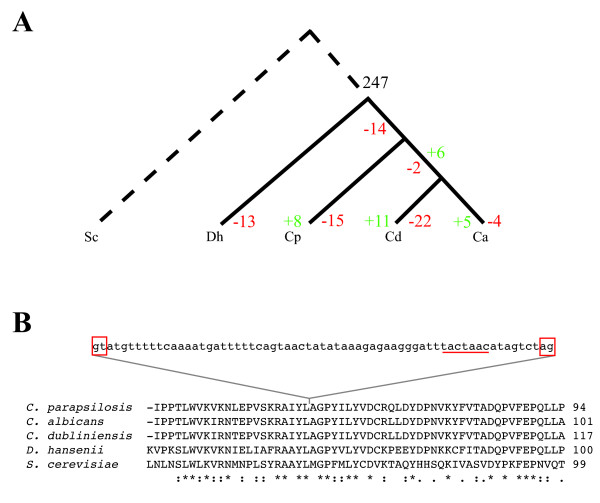
**Analysis of intron gain and loss in *Candida *species**. (A) 247 introns are conserved in four *Candida *species. We used *S. cerevisiae *and other species as outgroups to try to identify introns that were lost or gained in specific lineages. Losses are indicated in red and gains in green. Dh: *D. hansenii*; Cp: *C. parapsilosis*, Cd: C. *dubliniensis*, Ca: *C. albicans*. (B) Example of an intron gain in the *C. parapsilosis *ORF, Cpar2_502430. This intron is not present in any of the other four species.

We found that among 4869 orthologous genes present in all four species, 425 have introns in at least one species (466 introns in total, as some genes have more than one intron) (Additional file 7). We attempted to identify introns that were gained or lost in individual species using the approach described in Zhang *et al *[37] (Figure 3). An intron was scored as a "gain" if it was present in only one *Candida *species (or lineage) (Additional file 7). An intron is assumed to be "lost" if it is present in most of the species, and missing in either a single species or a single lineage. We could not determine the evolutionary origin of most introns (247), which are conserved in all the species tested. Other cases were ambiguous, for example introns present in only the orthologs from *C. dubliniensis *and *D. hansenii*, could represent independent gains in these species, or independent losses in the others. Where possible we included outgroups such as *Saccharomyces cerevisiae *and *Pichia pastoris *[38-40] to evaluate potential intron gains. Overall, we identified at least 30 cases of intron gain, and 70 cases of intron loss. We identified 68 introns present and 12 introns absent in *D. hansenii *genes only, which may result from intron gain/loss. However, we have not included this in Figure 3 because we are lacking well-annotated sequence information from close relatives of *D. hansenii *and so we cannot determine when the gain/losses occurred.

We identified eight instances where introns were apparently gained in *C. parapsilosis*, and 15 examples of intron loss (Additional file 7). Figure 3 shows one example of intron gain. At least 6 of these introns are also present in orthologs from *C. orthopsilosis *(Riccombeni *et al*, in preparation), and were therefore most likely gained in the common ancestor. We could not determine the presence of the remaining two in *C. orthopsilosis *because of a lack of sequence information. In most eukaryotes, intron loss is much more common than intron gain [41-44]. However, intron gain is relatively common in the Ascomycetes [37,45]. The molecular mechanisms are still not known, and are hypothesized to include insertion of transposable elements, insertion of spliced introns from other genes into mRNA followed by reverse transcription, and genomic duplication [41]. Intron loss is most likely to occur by reverse transcription of spliced messenger mRNAs [41]. The origin of the gained introns in *C. parapsilosis *is not clear; there are no orthologous sequences elsewhere in the nuclear or mitochondrial genome.

### Identification of novel transcribed regions

Analysis of the transcriptional profile of *C. albicans *using RNA-seq and microarrays has identified several hundred, and possibly several thousand novel transcribed regions (nTARs) that are unlikely to encode proteins [29-31]. We manually examined the *C. parapsilosis *data for evidence of nTARs. Because there is a wide variety in the number of novel transcribed regions identified by different methods [29-31], we used a very conservative manual approach, and counted only those *C. parapsilosis *regions where transcription is visually higher than background. We identified 95 regions that do not overlap with annotated ORFs (Additional file 8), and do not appear to encode proteins. We suspect that many of these regions may represent non-coding RNAs or other regulatory RNAs. In fact, some are small nuclear RNAs, such as the example shown in Figure 1C.

### Analysis of the transcriptional response of *C. parapsilosis *to hypoxia

Exposure of *C. albicans *to hypoxic conditions, such as those encountered in the host during infection, results in a switch from yeast-like to hyphal growth, increased expression of genes involved in ergosterol synthesis and glycolysis, and decreased expression of genes encoding steps in the TCA cycle and in oxidative phosphorylation [46-51]. *C. parapsilosis *does not undergo true hyphal growth. However, we previously used microarray profiling to show that similar metabolic changes (i.e. increases in glycolysis and ergosterol synthesis, decreases in respiration) occur in cells exposed to low oxygen in minimal media [18]. The transcriptional profile of cells growing in hypoxia resembles that of biofilm cells for both *C. albicans *and *C. parapsilosis*, and it has been shown that the metabolic adaptation to hypoxia is important for biofilm development by *C. albicans *[18,52-54].

We describe here the use of RNA-seq and microarray profiling to determine the transcriptional profile of *C. parapsilosis *cells grown in rich medium in hypoxic conditions. For the RNA-seq experiments, we observed a high correlation among six biological replicates grown in YPD in normoxia (1 > r > 0.95) and four biological replicates grown in hypoxia (0.96 > r > 0.82) (Additional file 9). Differentially expressed genes were identified by using Cufflinks, open source software specifically design to measure transcriptional differences [55]. Expression values were defined using FPKM and quantile normalization was applied.

**Figure 4:**
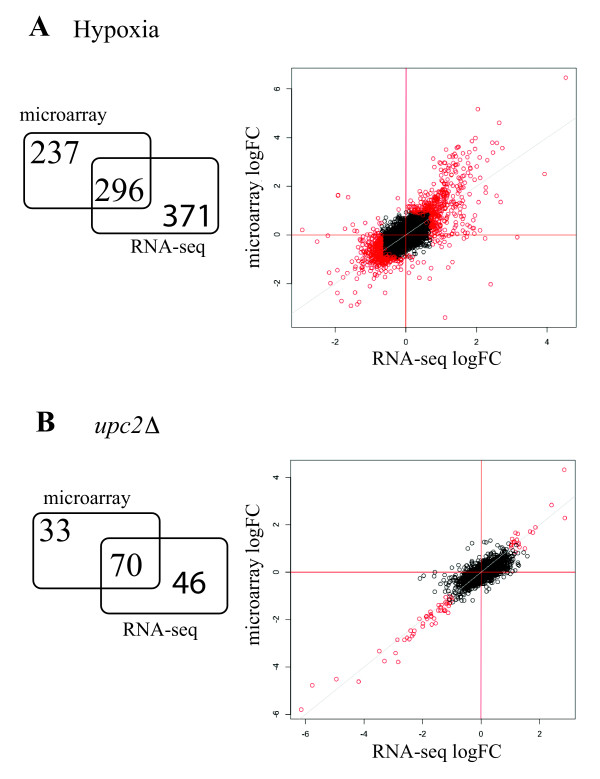
**Comparison of RNA-seq and array profiling**. (A) Correlation between the RNA-seq and microarray analysis for cells grown in rich media in hypoxic conditions. (B) Correlation between the RNA-seq and microarray analysis for the comparison of the *upc2 *deletion and wildtype cells grown in hypoxia. The log fold-changes of the FPKM ratio from the RNA-seq experiments is plotted on the x-axis, and the mean log fold-change from the microarray analysis is plotted on the y-axis. Each circle corresponds to a single gene; genes that are significantly differentially expressed in both microarray and RNA-seq analyses are colored in red.

A large number of genes are differentially expressed. Most are up-regulated in hypoxic conditions (347 genes up-regulated of 667 in total) (Additional file 10A). We identified more genes with altered expression using RNA-seq than by using microarrays [18] (667 versus 533, Additional file 10, Figure 4). However, although the microarray and RNA-seq profiling experiments were carried out at different times and by different personnel, the correlation between the two approaches is high (r = 0.73) (Figure 4). For example, we observed a high degree of overlap between the GO categories that are significantly enriched using the two approaches (Additional file 11). These include increased expression of genes involved in carbohydrate metabolism and glycolysis, fatty acid metabolism, and ergosterol biosynthesis, and decreased expression of genes involved in cellular respiration and the Tricarboxylic Acid Cycle (Additional file 11). For up-regulated genes all of the GO processes identified by microarray profiling are also present in the RNA-seq data set; some additional categories were identified in the RNA-seq profile only, including fatty acid oxidation and glucose transport (Additional file 11A). For the down-regulated genes, some categories such as amino-acid biosynthesis genes are over-represented in the microarray profiling experiments, and purine metabolism is over-represented in the RNA-seq data only. Some of the differences observed may reflect differences in experimental conditions; however, overall the two approaches produced very similar results.

The cpar2_404850 gene has the most dramatic increase in expression in the RNA-seq (5- fold) and in the microarray profiling experiments (6.5-fold) (Additional file 10). Cpar2_404850 has no known ortholog in other *Candida *species, no conserved domains, and no significant similarity to any other known proteins. Expression of cpar2_404850 is similar to that of the ergosterol pathway genes (see discussion of cluster 3 below), and will be the subject of future investigation. We have previously shown that *RBT1 *expression is increased during growth in minimal media in hypoxic conditions, and that the gene is required for optimal biofilm formation by *C. parapsilosis *[18]; our current analyses confirms that hypoxic induction of *RBT1 *also occurs during growth in rich media (Additional file 10).

**Figure 5:**
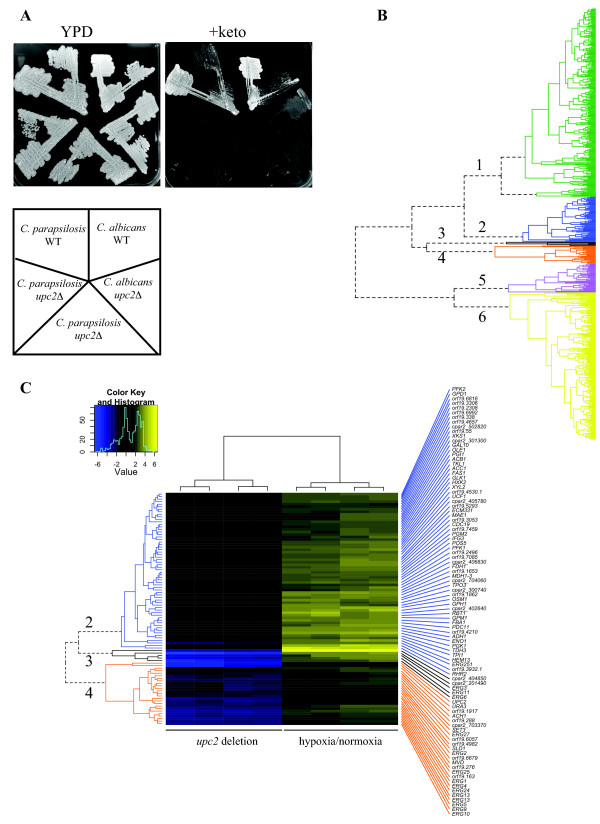
**Analysis of the *upc2 *deletion in *C. parapsilosis***. (A) Wildtype and *upc2 *deletion strains of *C. albicans *and *C. parapsilosis *were grown on YPD or YPD + 0.02 mg/ml ketoconazole plates for 5 days. Deleting *UPC2 *greatly increases the sensitivity of both species to ketoconazole. Strains: *C. albicans *WT (SC5314), *C. albicans upc2Δ *(TW14920), *C. parapsilosis *WT (CLIB214), and two independent knockouts of *upc2*in *C. parapsilosis *(CDupc5 and CDupc7). (B) Cluster analysis of genes differentially expressed in hypoxia and in the *upc2 *deletion of *C. parapsilosis*. Details of the clusters are provided in Additional file 15. (C) Clusters 2, 3 and 4 from (B). The names of the *C. albicans *orthologs are given where available. Where there is no ortholog, the cpar2 gene name is shown.

In *S. cerevisiae*, expression of oxygen-responsive genes in controlled by monitoring the levels of heme and ergosterol [56-59]. Ergosterol synthesis genes are regulated by the paralog pair *ECM22 *and *UPC2*, which respond to sterol levels [60]. *C. albicans *has only one ortholog of *ECM22 *and *UPC2 *(called *UPC2*), and this is required for the up-regulation of ergosterol and drug resistance genes in hypoxia [22,23,51,61]. We therefore determined the effect of knocking out the ortholog of *UPC2 *in *C. parapsilosis*. One allele was knocked out by replacing *HIS1*, and one by replacing with *URA3 *(Additional file 12). Knocking out *UPC2 *increases the sensitivity of *C. parapsilosis *cells to azole drugs (Figure 5), similar to the phenotype reported for *C. albicans *[23,61]. We used both microarrays and RNA-seq to determine the transcriptional profile of wildtype and *upc2 *deletion cells grown in hypoxic conditions. We identified 116 genes that are differentially expressed in the *upc2 *deletion grown using RNA-seq, and 103 genes using microarray profiling (Additional file13A, B). The same cell cultures were used for both experiments, and there is a high correlation between the gene expression levels identified by the two methods (r = 0.84, Figure 4B). 70 genes with differential expression were identified by both methods (Additional file 13C). GO analysis indicates that genes involved in ergosterol biosynthesis, lipid biosynthesis and response to drug have decreased expression in the *upc2 *deletion (Additional file 14).

To assess the importance of Upc2 as an hypoxic regulator, we used hierarchical cluster analysis to identify genes with shared expression patterns in the hypoxia/normoxia dataset and in the *upc2 *deletion (Figure 5). We used the profiles generated from microarrays rather than RNA-seq for this comparison, partly because the methods are better developed, and because it allowed us to compare our results with our previously published analysis of the hypoxic response of *C. albicans *[51]. We identified six major clusters (Figure 5B, Additional file 15). Expression of genes in clusters 3 (5 genes) and 4 (25 genes) is greatly increased in wildtype cells grown in hypoxia, and is repressed in the *upc2 *deletion grown in the same conditions (Figure 5C). These clusters contain most of the genes required for ergosterol metabolism. Cluster 3 includes 3 genes encoding oxygen-dependent enzymes (*ERG3, ERG6 *and *ERG11*) whereas cluster 4 includes both oxygen-dependent (*ERG1, ERG2, ERG4, ERG5, ERG24, ERG25, ERG27*) and oxygen-independent steps (*MVD, ERG9, ERG10, ERG13*) (Figure 5C, Additional file 15). Genes in cluster 2 are also induced in the wildtype in hypoxia, but not in the *upc2 *deletion. GO analysis indicated that this cluster is enriched in processes associated with glycolysis and carbohydrate metabolism (Additional file 15B). Genes in cluster 1 are induced in hypoxia in wildtype and in the *upc2 *deletion (enriched in processes associated with oxidation-reduction), and genes in cluster 6 have reduced expression in both strains (enriched in processes associated with the TCA cycle, cellular respiration and ATP synthesis). Genes in cluster 5 are more upregulated in hypoxia in the *upc2 *deletion, and are not enriched for any specific GO processes

Our results suggest that Upc2 in *C. parapsilosis *is required for induction of expression of ergosterol synthesis genes in hypoxia (clusters 3 and 4). In the absence of *UPC2*, expression of these genes is repressed. Deleting *UPC2 *decreases the hypoxic induction of glycolytic genes (cluster 2), but not to as great an extent as the reduction in expression of the ergosterol pathway. It is therefore likely that similar to *C. albicans*, expression of the ergosterol pathway in *C. parapsilosis *is directly regulated by Upc2, but expression of glycolytic genes although influenced by Upc2 is likely to be controlled by other transcription factors (such as Gal4 and Tye7, as in *C. albicans *[46]). Upc2 does not play any role in the reduction of expression of respiratory genes in hypoxia (cluster 6) nor in the induction of genes associated with oxidation-reduction (cluster 1). The potential role of Upc2 to regulate genes in cluster 5 requires further investigation.

We did not observe a statistically significant enrichment of iron metabolism genes during hypoxic growth of *C. parapsilosis*, in either the microarray or the RNA-seq data. This differs from *C. albicans *[51]. However, expression of at least three ferric reductases (*FRE10*, *FRP1 *and *CFL5*), an iron permease (*FTR1*), a multicopper oxidase and several iron-containing proteins is decreased, and expression of *HEM13*, heme oxygenase (*HMX1*) and a catalase (*CAT1*) is increased in the RNA-seq data, and for some also in the microarray data (Additional file 10). It is therefore likely that iron metabolism genes are similarly regulated in the two species. Overall, our analyses indicate that *C. albicans *and *C. parapsilosis *have a similar response to hypoxia, and that the role of Upc2 is conserved.

## Conclusions

We describe the first comprehensive annotation of the *C. parapsilosis *genome, which is supported by expression analysis. We identified > 300 new open reading frames and corrected the annotation of hundreds more. We also identified several nTARs, many of which are likely to represent non-coding RNAs (ncRNA). We identified two introns in the 3' UTR of a single gene, suggesting that regulated splicing may be involved in gene regulation. *C. parapsilosis *genes have few introns, similar to other species in the Saccharomycotina. However, we identified a small number of introns that were both gained and lost in recent evolutionary history. We also used RNA-seq and microarray profiling to identify the targets of the Upc2 transcription factor, and show that it has a major role as a regulator of ergosterol synthesis in *C. parapsilosis*.

## Methods

### Assembly

The *C. parapsilosis *genome was originally assembled as described in Butler *et al *[15] and submitted to EMBL as 24 contigs (CABE01000001-CABE01000024). Some errors and gaps have now been manually corrected, and the genome has now been assembled into 8 chromosome-sized contigs larger than 200 kb (version 012609). We have re-named the major contigs as chromosomes 1 (contig005569), 2 (contig005809), 3 (contig005806), 4 (contig006372), 5 (contig006139), 6 (contig005504), 7 (contig006110) and 8 (contig005807). The mitochondrial genome is represented by contig006121. The assembly has replaced the original submission, and is available at accession numbers HE605202-HE605210.

### Strains and Media

The strains used in this study are listed in Additional file 16. Oligonucleotide primers used in RT-PCR and to validate constructs and introns are listed in Additional file 17. Cells were grown in YPD (1% Yeast Extract, 2% Peptone, 2% Glucose; FormediumTM), YPGlycerol (YPD with 2% glycerol instead of 2% glucose), BMW (1.5% Yeast Extract, 1% Peptone, 2% Glucose, 0.2% SC Amino Acid mix, 0.01% Adenine, 0.01% Tryptophan, 0.01% Uracil; [62,63]) and synthetic defined (SD) media (0.676% Yeast Nitrogen Base without amino acids, 2% Glucose). Cells were grown at 30°C and 21% O_2 _unless otherwise noted. Where indicated, cells were grown in 1% oxygen, 99% nitrogen in an InVivo_2 _400 hypoxic chamber.

### Generating a knockout of the *C. parapsilosis UPC2 *gene

Both alleles of *UPC2 *were knocked out by replacement with *URA3 *and *HIS1 *in *C. parapsilosis CDUhis11 *(Additional file 12). The *URA3 *or *HIS1 *disruption cassettes were generated from plasmid pLUL2 or pLHL2 [64], respectively using oligonucleotides, UPCUH_F and UPCUH_R (Additional file 17).

### RNA isolation

The cells were harvested by centrifugation and either subjected to RNA extraction or frozen at -80°C. Total RNA was extracted from fresh or frozen cell pellets using a RiboPure Yeast Kit (Ambion). RNA concentrations were determined using a NanoDrop 1000 (Thermo Scientific), while quality and integrity was checked using a Bioanalyzer 2100 (Agilent Technologies).

### Microarray analysis

The hypoxia experiments in rich media were carried out at the same time as our previous analysis of the hypoxic response in minimal media [18]. However the results were not reported previously. Overnight cultures of *C. parapsilosis *CLIB214 were subcultured in 300 ml YPD at an A_600 _of 0.2. The culture was maintained at 30°C for 3 h at atmospheric oxygen conditions. Two aliquots were then removed after 3 h cell growth; one culture was incubated at atmospheric oxygen conditions, and the other was incubated in 1% O_2 _for an additional 3 h. RNA was isolated and cDNA generated and labeled with Cy3 or Cy5 as described in Rossignol *et al *[18]. Four biological replicates were compared: in all four the normoxic samples were labeled with Cy3 and the hypoxic samples were labeled with Cy5. To determine the transcriptional profile of the *upc2 *deletion, *C. parapsilosis *CLIB214 (wildtype) and *C. parapsilosis *CDupc5 (*upc2 *deletion) cells were grown in YPD at atmospheric oxygen for 3.5 h and then transferred to 1% O_2 _for an additional 2 h. Four biological replicates were used: in the first two, RNA from the wildtype strain was labeled with Cy3 and the *upc2 *deletion was labeled with Cy5. cDNA was generated, labeled, and hybridized to *C. parapsilosis *microarrays designed and manufactured using eArray from Agilent as described in Rossignol *et al *[18]. The microarray platform is described on the NCBI Gene Expression Omnibus Database (GEO), with the ID GPL13192. Data were analyzed using the Limma package [65] from Bioconductor http://bioconductor.org as described in [18,51]. Only genes with a fold change (FC) greater than 2 and an adjusted *p*-value less than 0.05 were considered.

### GO enrichment analysis

All the GO term enrichment analyses were performed using the web application "GO term finder" available on the "Candida Genome Database" (CGD, http://www.candidagenome.org/cgi-bin/GO/goTermFinder). When testing *C. parapsilosis *gene lists, the *C. albicans *orthologs extracted from the Candida Gene Order Browser [19] CGOB were used, and the background for the test was appropriately adjusted by excluding those *C. albicans *genes found not to have a *C. parapsilosis *ortholog.

### RNA-seq library preparation

Cells were grown at 30°C overnight and then diluted to an A_600 _of 0.2, and grown for an additional 5 h at 30°C or 37°C in YPD, BMW, YPglycerol or SD media in either 21% or 1% O_2 _(Additional file 1). mRNA purified from total RNA using oligo dT Dynabeads (Invitrogen) was fragmented to an average size of 200 nucleotides by a 5 minute heat treatment (70°C) with a buffered zinc solution (Fragmentation Reagent, Ambion). Fragmentation of mRNA was stopped using an EDTA based Stop buffer (Ambion). Fragmented mRNA was incubated with 3 μg Random Hexamer Primers at 65°C for 5 min. First strand cDNA synthesis was carried out using 1 × First Strand Buffer (Invitrogen), 10 mM DTT, 500 μM dNTP mix (Invitrogen), 20 Units RNaseOUT and 200 units SuperScript™ II Reverse Transcriptase (Invitrogen). For strand-specific library generation, unincorporated dNTPs were subsequently removed using G-50 Micro Columns (GE Healthcare). Second strand cDNA was generated using 1× Second Strand Buffer, 300 μM dNTP mix, 2 units RNaseH, 50 units DNA Polymerase I (NEB), while a 300 μM dUTP mix was used instead of a dNTP mix for the generation of a strand specific library. This material was used for library preparation. The method for strand-specific library preparation was adapted from Parkhomchuk *et al *[66] as described in Weissenmayer *et al *[67]. Briefly, DNA fragments were blunted in an End Repair reaction using T4 DNA Polymerase, Klenow DNA Polymerase and T4 Polynucleotide Kinase, after which a single 'A' base was added to the 3' end using dATP and Klenow Exo Fragment. Illumina adapters were ligated to the ends of the DNA fragments, allowing for the subsequent hybridization to a flow cell. Fragments of approximately 300 base pairs were purified from a 2% Agarose Gel. For the strand-specific libraries, the second strand containing uridine was removed by treatment with 1 unit Uracil N-glycosylase (UNG) in TE Buffer at 37°C for 15 min. In all cases, purified adapter ligated DNA templates were then amplified through PCR enrichment, using PCR primers (Illumina), a dNTP mix, Phusion Polymerase (NEB) and 16 cycles of PCR. All libraries were quantified using a Qubit Fluorometer (Invitrogen) and assessed on a 2% agarose gel. Amplified libraries were loaded on a flow cell. Sequencing was carried out in-house by running at least 36 cycles on an Illumina Genome Analyzer IIx according to manufacturer's instructions, resulting in read lengths of approximately 42 bases. In addition, long reads (76 bases) were generated from two libraries by GATC Biotech AG using an Illumina Genome Analyzer IIx (Additional file 1).

### Read mapping and expression analysis

In-house reads were processed according to version 1.4 of Illumina's Genome Analysis Pipeline. FastQ files were preprocessed by customized scripts designed to control data quality and to detect the presence of adapter sequences (data not shown). Reads from each dataset were aligned to the 8 *C. parapsilosis *chromosomal contigs using TopHat [25]. To minimize mapping errors and address the issue of repetitive regions, reads which mapped to more than one location or with more than 2 alignment mis-matches were discarded. TopHat was first set to detect introns with length not lower than 70 nucleotides and, secondly with length not lower than 10 nucleotides. The results were handled using SAMtools, a package of computational tools for next generation sequencing format manipulation [68]. Reads that mapped to the genome through a gapped alignment were identified and isolated. Each candidate was manually curated and discarded where necessary. Data were visualized using the Artemis genome browser [26] and Tablet, a next generation sequence assembly visualization tool [69].

The RNA-seq data was combined with the existing cpag and cpar annotations [15,18] and mapped onto the 8 chromosome contigs. Genes are named using the prefix "Cpar2". Gene models are numbered using a six-digit number; the first digit specifies the chromosome. 91 Putative tRNA genes were identified and were incorporated into the annotation using tRNAscan-SE [27].

We applied CuffDiff from the package Cufflinks [55] to identify differentially expressed genes. Cufflinks measures FPKM (fragments per kilobase of transcript per million fragments mapped); the results of the differential expression test are reported as log ratios of the FPKM values. CuffDiff incorporates data from biological repeats (listed in additional File 1). We used 4 biological replicates grown in hypoxic condition and 5 biological replicates from normoxic conditions (libraries generated with longer reads and technical replicates where not included); and 4 biological replicates from wildtype and 2 biological replicates from a *upc2 *deletion grown in hypoxic conditions. Quantile-based normalization was used to identify differentially expressed genes [70]. Genes with a *p*-value of < 0.05 were considered to be differentially expressed. A Benjamini-Hochberg [71] correction for multiple-testing was also applied and genes with *p*-value greater then the FDR were discarded.

Some minor annotation changes were made to the genome after most of the analyses (expression analysis, UTR identification etc.) were carried out. The most up-to-date annotation was submitted to EMBL, and is included in Table 1.

### UTR detection

To identify the 5' and 3' UTR boundaries of protein-coding genes, we scanned the RNA-seq coverage from each experimental condition. The coverage upstream from the ATG and downstream from the stop codon of each annotated ORF was examined by an in-house program which counted the number of reads mapped at each coordinate. The longest continuous signal with at least 2 reads at each coordinate was considered. If the signal extended as far as reaching the boundary of the adjacent gene in at least one experiment, the region was considered to be "overlapping" and therefore impossible to resolve. Strand-specific data were used to map the UTRs of genes transcribed from different strands. Regions with low expression or with lack of RNA-seq evidence were discarded and marked with "no coverage". A special tag was added to those genes near the edges of the contig; in cases where the UTR signal extended to the last or first coordinate the regions was annotated as "reached end of the contig". Genes with introns in the 5' and 3' regions were manually corrected (Additional file 5).

### nTAR identification

nTARs (novel transcriptional active regions) were identified during the manual curation of the genome. The overall expression was visualized with the Artemis genome annotation browser [26] for each biological condition. Whenever it was not possible to identify an open reading frame, or where any potential ORFs were < 100 amino acids and not conserved in other *Candida *species, we marked the location as a possible nTAR.

### Intron loss/gain analysis

Orthologous groups of genes from four species (*C. albicans, C. dubliniensis, C. parapsilosis *and *D. hansenii*) were identified using CGOB (Maguire *et al*, in preparation) [19]. Genes containing at least one intron in one species were identified. The orthologous proteins were aligned using T-Coffee [72]. A PERL script was written to identify introns conserved in 2 or more species, defined as being within 10 amino acids in the multiple sequence alignment. Where possible, orthologous genes were identified in outgroup species including *S. cerevisiae*, *Pichia pastoris*, *Lachancea kluyveri *and *Eremothecium gossypii*. Intron gain/loss was determined by manual inspection (Additional file 7). For example, introns present in *C. albicans *and *C. dubliniensis *only were assumed to be gained on this branch, rather than lost in all other species. For several cases it was not possible to determine if specific introns were gained or lost. We noticed that one gene in *C. dubliniensis *(CD36_81540) and one in *D. hansenii *(DEHA2G18062g) contain putative introns that are not in the published annotations.

### Accession codes and Supplementary Websites

Raw microarray data and the description of the array have been deposited in the Gene Expression Omnibus Database, under the series number GSE32716. The annotation is available from EMBL (accession no HE605202-HE605210).

## Abbreviations

CGD: *Candida *Genome Database; CGOB: *Candida *Gene Order Browser; FPKM: fragments per kilobase of transcript per million fragments mapped; GO: Gene Ontology; ncRNA: non-coding RNA; nTAR: novel transcriptional active region; ORF: open reading frame; RT: reverse transcription; snRNA: small nuclear RNA; UTR: untranslated region.

## Competing interests

The authors declare that they have no competing interests.

## Authors' contributions

GB, AG and CL conceived and designed the experiments. NC and MB sequenced and assembled the genome. CL carried out the RNA-seq and microarray experiments. AG mapped and analyzed the data, with contributions from DH. SM identified tRNAs and analyzed the loss and gain of introns. CD generated the *UPC2 *deletion. GB and AG wrote the paper, with contributions from CL and SM. All authors read and approved the final manuscript.

## Supplementary Material

Additional file 1 **Experimental conditions used for RNA-seq analysis**. List of conditions used in RNA-seq experiments. Click here for file

Additional file 2 **Analysis of UTR regions in *C. parapsilosis *genes**. Table showing the results of the UTR discovery analysis. Click here for file

Additional file 3 **GO term enrichment analysis of genes with 5'UTR longer than 500 bp**. GO term enrichment analysis performed on genes with 5' UTR longer than 500 bp. Click here for file

Additional file 4 **Identification of introns in *C. parapsilosis***. File listing all the introns and the gene structure with corresponding coordinates. Click here for file

Additional file 5 **Validation of selected introns**. The table shows the expected product sizes from genomic DNA and from cDNA, as well as information about the *C. albicans *orthologs. Click here for file

Additional file 6 **Intron consensus sequences and length distribution**. (A) Comparison of intron length between *C. parapsilosis *and *C. albicans*. (B) Comparison of splice-site conservation between *Candida parapsilosis *and *Candida albicans*. Click here for file

Additional file 7 **Intron conservation in *Candida *species**. Conservation of introns in four Candida genomes (*C. albicans*, *C. dubliniensis*, *C. parapsilosis *and *D. hansenii*). Click here for file

Additional file 8 **Identification of novel transcriptional active regions (nTARs)**. Transcribed regions that do not overlap with annotated ORFs and do not appear to encode proteins. Click here for file

Additional file 9 **Correlation between data from biological replicates for RNA-seq**. (A) Heatmap plot showing the correlation of the raw FPKM from the different RNA-seq conditions. (B) Boxplot of the total FPKM values for each RNA-seq library. Click here for file

Additional file 10 **Genes differentially expressed in *C. parapsilosis *in normoxic versus hypoxic conditions**. List of genes differentially expressed in hypoxia versus normoxia from (A) RNA-seq and (B) microarray experiment. Click here for file

Additional file 11 **GO term analysis of genes differentially expressed in hypoxia from RNA-seq and microarray profiling**. (A) Comparison of enriched GO terms from RNA-seq and microarray profiling (B) GO terms enriched in up-regulated genes from microarrays (C) GO terms enriched in down-regulated genes from microarrays (D) GO terms enriched in down-regulated genes from RNA-seq (E) GO terms enriched in up-regulated genes from RNA-seq. Click here for file

Additional file 12 **Generating a gene knockout of *UPC2 *in *C. parapsilosis***. Generating a gene knockout of *UPC2 *in *C. parapsilosis*. Click here for file

Additional file 13 **Genes that are differentially regulated in a *C. parapsilosis upc2 *deletion in hypoxic conditions, identified by both microarrays and RNA-seq**. (A) Gene found to be differentially expressed using RNA-seq (B) Genes differentially expressed using microarrays (C) Genes differentially expressed in both RNA-seq and microarray experiments. Click here for file

Additional file 14 **GO term enrichment analysis of genes differentially expressed in an *upc2 *deletion**. A) GO term enrichment analysis of genes down-regulated in an *upc2 *deletion B) GO term enrichment analysis of genes up-regulated in an *upc2 *deletion. Click here for file

Additional file 15 **Cluster analysis of genes differentially expressed in hypoxia in wildtype and in the *upc2 *deletion**. Hierarchical clustering of differentially expressed genes in wildtype and *upc2 *deletion of *C. parapsilosis *grown in hypoxia. Click here for file

Additional file 16 **Strains used in this study**. List of the strains used in this study. Click here for file

Additional file 17 **Oligonucleotide primers**. List of oligonucleotide primers used in RT-PCR and to validate constructs and introns. Click here for file
