# Supplementary material for: Using RNA-seq to determine the transcriptional landscape and the hypoxic response of the pathogenic yeast Candida parapsilosis
Source: BMC Genomics. 2011 Dec 22;12:628. doi: 10.1186/1471-2164-12-628 (PMC3287387; doi:10.1186/1471-2164-12-628)
Supplement: Additional file 1 — Experimental conditions used for RNA-seq analysis. List of conditions used in RNA-seq experiments. [file 1471-2164-12-628-S1.DOC]

# **Additional file 1. Experimental conditions used for RNA-seq analysis**

| Growth Condition^(1)^ | nt^(2)^ | ss^(3)^ | tot. reads | aligned reads | rRNA^(4)^ |
| --- | --- | --- | --- | --- | --- |
| BMW, 30ºC #1 | 78 |  | 5,585,130 | 3,378,849 (60.5%) | 11,199 (0.3%) |
| BMW, 30ºC #2 | 42 |  | 11,101,586 | 9,874,324 (88.9%) | 970,583 (9.8%) |
| BMW, 30ºC #2 | 42 |  | 11,923,773 | 10,670,375 (89.5%) | 885,851 (8.3%) |
| BMW, 30ºC #3 | 42 |  | 14,683,862 | 13,112,569 (89.3%) | 758,259 (5.8%) |
| BMW, 30ºC #3 | 42 |  | 11,457,771 | 9,472,924 (82.7%) | 438,887 (4.6%) |
| BMW, 30ºC #4 | 42 |  | 14,372,512 | 12,929,788 (90.0%) | 966,255 (7.5%) |
| BMW, 30ºC #4 | 42 |  | 14,314,337 | 12,750,338 (89.1%) | 785,952 (6.2%) |
| YPD, 30ºC #1 | 78 |  | 8,253,239 | 5,309,347 (64.3%) | 4,263 (0.08%) |
| YPD, 30ºC #2 | 42 |  | 13,442,123 | 11,992,257 (89.2%) | 1,238,331 (10.3%) |
| YPD, 30ºC #2 | 42 |  | 12,523,951 | 11,048,946 (88.2%) | 919,727 (8.3%) |
| YPD, 30ºC #3 | 42 |  | 15,579,613 | 13,822,912 (88.7%) | 956,507 (6.9%) |
| YPD, 30ºC #3 | 42 |  | 10,213,665 | 6,606,721 (64.7%) | 387,196 (5.9%) |
| YPD, 30ºC #4 | 42 |  | 17,071,805 | 15,202,089 (89.0%) | 1,005,631 (6.6%) |
| YPD, 30ºC #4 | 42 |  | 11,848,266 | 9790774 (82.6%) | 554,722 (5.7%) |
| YPD, 30ºC #5 | 42 |  | 15,588,509 | 13,877,893 (89.0%) | 1,139,267 (8.2%) |
| YPD, 30ºC #5 | 42 |  | 12,397,827 | 10,063,357 (81.2%) | 684,829 (6.8%) |
| YPD, 30ºC #6 | 42 | ✓ | 16,378,297 | 12,916,991 (78.9%) | 1,808,303 (14.0%) |
| YPD, 30ºC #7 | 42 | ✓ | 18,927,555 | 17,335,221 (91.6%) | 2,721,541 (15.7%) |
| YPD, 30ºC, 1% O_2_  #1 | 42 |  | 11,634,551 | 10,376,831 (89.2%) | 1,497,691 (14.4%) |
| YPD, 30ºC, 1% O_2_  #2 | 42 | ✓ | 6,223,791 | 4,830,036 (77.6%) | 704,382 (14.6%) |
| YPD, 30ºC, 1% O_2_  #3 | 42 | ✓ | 10,136,009 | 8,781,619 (86.6%) | 852,511 (9.7%) |
| YPD, 30ºC, 1% O_2_  #4 | 42 | ✓ | 12,557,342 | 11,162,720 (88.9%) | 820,086 (7.3%) |
| YPD, 37ºC #1 | 42 |  | 12,874,029 | 11,337,532 (88.1%) | 2,209,815 (19.5%) |
| YPD, 30ºC, 1% O_2_ , *upc2D*  #1 | 42 | ✓ | 8,978,940 | 6,909,418 (77.0%) | 909,325 (13.2%) |
| YPD, 30ºC, hypoxia, *upc2D*  #2 | 42 | ✓ | 22,373,115 | 20,665,206 (92.4%) | 1,920,026 (9.3%) |
| Mixture of media, 30ºC, 37ºC #1 | 42 |  | 11,008,547 | 9,714,988 (88.2%) | 1,139,820 (11.7%) |
| total |  |  | 331,450,145 | 283,934,025 (85.7%) | 26,290,959 (9.3%) |

^(1)^ Media, temperature and oxygen concentrations. Unless indicated, cultures were grown in 21% O_2_. Biological replicates are indicated using different number,s technical replicates have the same label number,

^(2)^ Length of sequence read (nt)

^(3)^ Checked when a strand specific protocol was used

^(4)^ Percentage of reads mapped to the ribosomal cluster.
