# Supplementary material for: Using RNA-seq to determine the transcriptional landscape and the hypoxic response of the pathogenic yeast Candida parapsilosis
Source: BMC Genomics. 2011 Dec 22;12:628. doi: 10.1186/1471-2164-12-628 (PMC3287387; doi:10.1186/1471-2164-12-628)
Supplement: Additional file 2 — Analysis of UTR regions in C. parapsilosis genes. Table showing the results of the UTR discovery analysis. [file 1471-2164-12-628-S2.DOC]

# **Additional file 2: Analysis of UTR regions**

| 5’ UTR | No. of genes |
| --- | --- |
| length > 100 bp | 2353 |
| length > 250 bp | 1049 |
| length > 500 bp | 295 |
| overlapping | 835 |
| reached contig extremities* | 3 |
| no coverage** | 295 |
| null*** | 219 |
| Total | 4699 |
| 3’ UTR |  |
| length > 100 bp | 1887 |
| length > 250 bp | 584 |
| length > 500 bp | 138 |
| overlapping | 2080 |
| reached contig extremities* | 6 |
| no coverage** | 195 |
| null*** | 221 |
| Total | 3549 |

* reached the end of the contig: UTRs that could not be identified because they extend beyond the end of the contig sequence.

** no coverage: Number of genes with no UTR annotation due to lack of RNA-seq coverage

***null : features that were not used to identify UTRs (e.g. tRNAs).
