# Supplementary material for: Using RNA-seq to determine the transcriptional landscape and the hypoxic response of the pathogenic yeast Candida parapsilosis
Source: BMC Genomics. 2011 Dec 22;12:628. doi: 10.1186/1471-2164-12-628 (PMC3287387; doi:10.1186/1471-2164-12-628)
Supplement: Additional file 5 — Validation of selected introns. The table shows the expected product sizes from genomic DNA and from cDNA, as well as information about the C. albicans orthologs. [file 1471-2164-12-628-S5.DOC]

# **Additional file 5: Validation of selected introns (see Figure 2).**

| **Cpar2 ID** | **Intron type** | ***C. albicans* ID** | ***C. albicans* gene name** | **Intron annotated *C. albicans*** | **Expected genomic DNA PCR product size (bp)** | **Expected cDNA PCR product size (bp)** |
| --- | --- | --- | --- | --- | --- | --- |
| Cpar2_601470 | 3’ UTR | orf19.3481 | uncharacterized |  | 471 | 315 |
| Cpar2_601470 | 3’ UTR | orf19.3481 | uncharacterized |  | 727 | 351 |
| Cpar2_601830 | internal | orf19.3504 | RPL23A | Yes | 680 | 334 |
| Cpar2_603230 | internal | orf19.114 | uncharacterized | No | 463 | 403 |
| Cpar2_807560 | internal | orf19.5927 | RPS15 | Yes | 521 | 240 |
| Cpar2_803400 | internal | orf19.6265.1 | RPS14B | Yes | 690 | 335 |
| Cpar2_400980 | 5’ UTR | orf19.4632 | RPL20B | No | 392 | 196 |
| Cpar2_205880 | 5’ UTR | orf19.6415.1 | RPS29A | No | 482 | 105 |
| Cpar2_209350 | internal | orf19.2311 | RPL82 | Yes | 374 | 145 |
