# Supplementary material for: Using RNA-seq to determine the transcriptional landscape and the hypoxic response of the pathogenic yeast Candida parapsilosis
Source: BMC Genomics. 2011 Dec 22;12:628. doi: 10.1186/1471-2164-12-628 (PMC3287387; doi:10.1186/1471-2164-12-628)
Supplement: Additional file 6 — Intron consensus sequences and length distribution. (A) Comparison of intron length between C. parapsilosis and C. albicans. (B) Comparison of splice-site conservation between Candida parapsilosis and Candida albicans. [file 1471-2164-12-628-S6.PDF]

A

C. albicans

5 prime

A	0.00	0.00	0.98	0.06	0.01	0.02
C	0.00	0.00	0.00	0.08	0.00	0.01
G	1.00	0.00	0.01	0.01	0.99	0.00
T	0.00	1.00	0.01	0.85	0.00	0.97

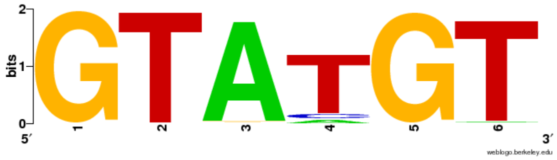

3 prime

A	0.32	0.30	0.20	0.42	0.15	1.00	0.00
C	0.09	0.12	0.07	0.16	0.13	0.00	0.00
G	0.10	0.10	0.06	0.04	0.00	0.00	1.00
T	0.49	0.48	0.67	0.38	0.72	0.00	0.00

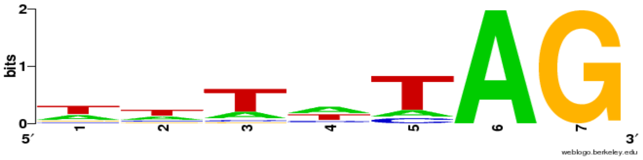

C. parapsilosis

5 prime

A	0.00	0.00	0.97	0.06	0.01	0.02
C	0.00	0.00	0.00	0.11	0.01	0.02
G	1.00	0.00	0.01	0.01	0.97	0.00
T	0.00	1.00	0.02	0.82	0.01	0.96

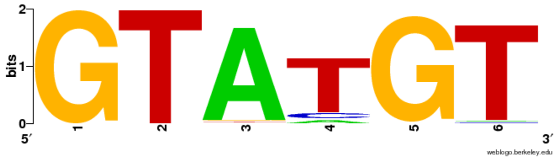

3 prime

A	0.26	0.22	0.18	0.27	0.11	0.99	0.00
C	0.20	0.24	0.17	0.29	0.21	0.00	0.00
G	0.08	0.08	0.04	0.09	0.00	0.01	1.00
T	0.46	0.46	0.61	0.35	0.68	0.00	0.00

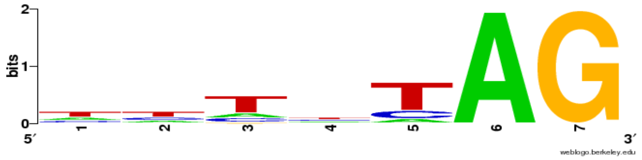

B

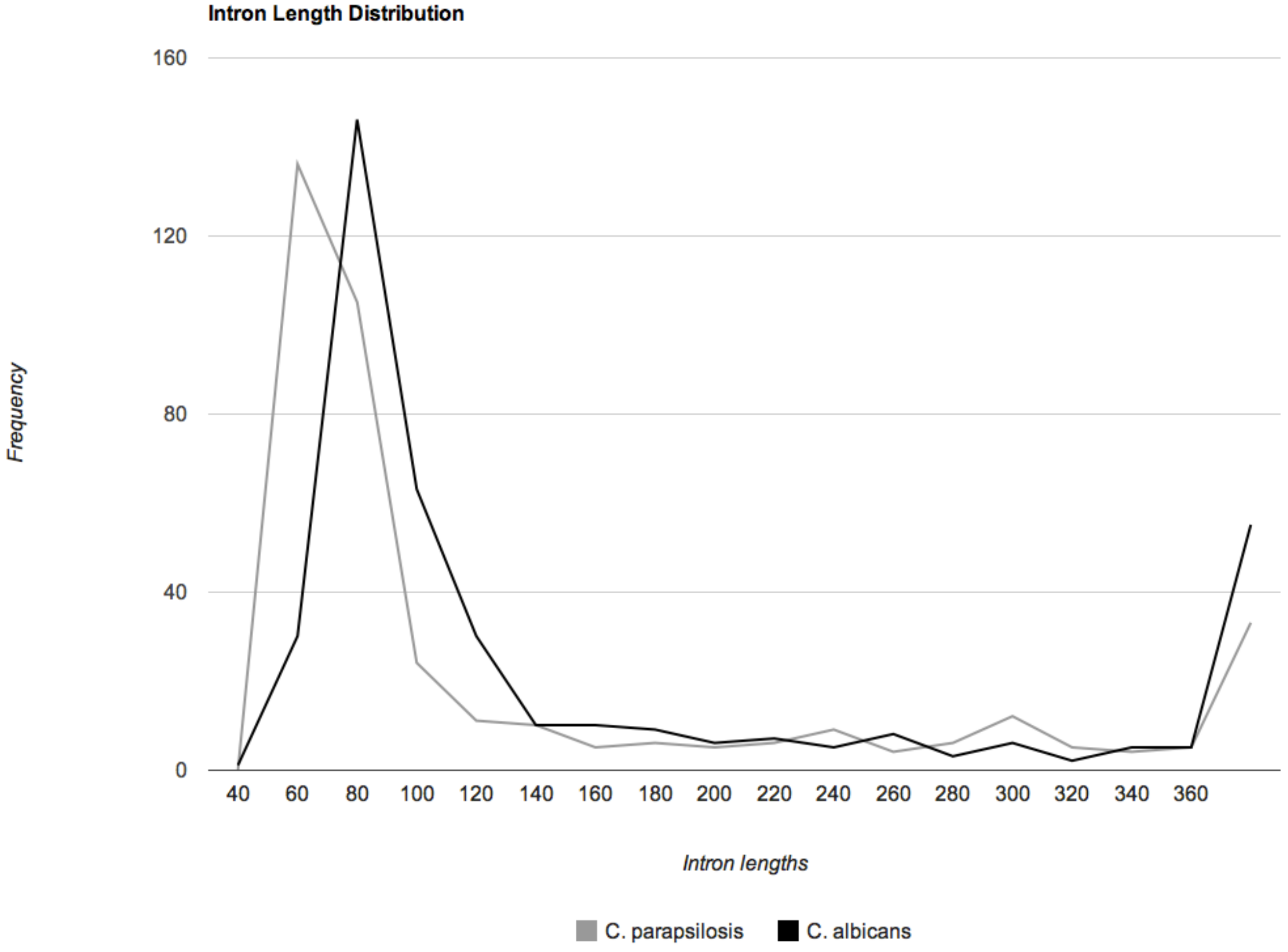

(A) Intron length comparison between C. parapsilosis and C. albicans. (B) Intron consensus sequence comparison between Candida parapsilosis and Candida albicans. The frequency matrixes were calculated from the intron sequences according to the C. albicans and C. parapsilosis latest annotation. The motif logo were generated by using the webtool http://www.benoslab.pitt.edu/stamp/index.php
