# Supplementary material for: Using RNA-seq to determine the transcriptional landscape and the hypoxic response of the pathogenic yeast Candida parapsilosis
Source: BMC Genomics. 2011 Dec 22;12:628. doi: 10.1186/1471-2164-12-628 (PMC3287387; doi:10.1186/1471-2164-12-628)
Supplement: Additional file 9 — Correlation between data from biological replicates for RNA-seq. (A) Heatmap plot showing the correlation of the raw FPKM from the different RNA-seq conditions. (B) Boxplot of the total FPKM values for each RNA-seq library. [file 1471-2164-12-628-S9.DOC]

### Additional file 9. Correlation between biological replicates for RNA-seq data sets.

###
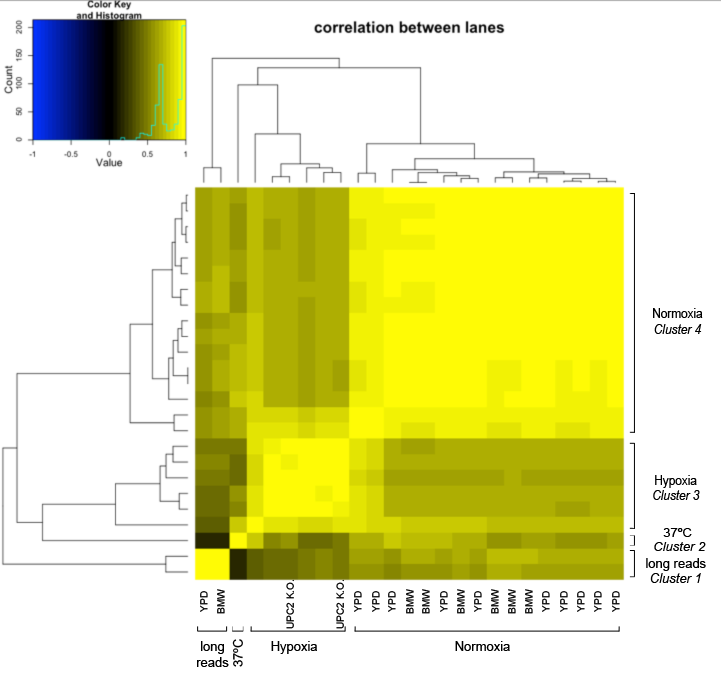


### (A) Correlation between biological replicates

Heatmap plot showing the correlation of the raw FPKM from the different RNA-seq conditions. Four main groups can be easily identified. The first cluster contains biological replicates generated with longer reads library (78bp). These two libraries were sequenced by GATC and are strongly correlated with each other (r=0.98) and weakly correlated (0.60<r<0.73) with all the other biological samples. These libraries were used for transcriptome mapping but not for analysis of differential gene expression. Cluster 2 is composed of one library only, generated from cells grown at 37ºC. This cluster does not correlate well with the other experimental conditions (0.15<r<0.80), and was not used in the expression analysis. Cluster 3 is obtained from cells grown in normoxic condition (r>0.93). There is no discrimination between cells grown in BMW and YPD media. Cluster 4 groups libraries made in hypoxic condition (r> 0.82). This group includes libraries generated from strains with an *upc2* deletion.


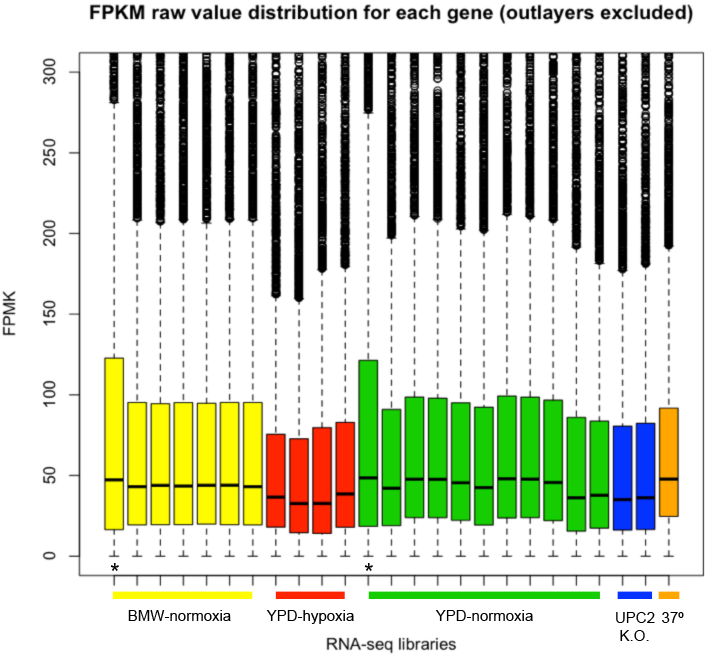


### (B) Distribution of the raw FPKM value among all the genes of the genome, excluding the outliers (>300 FPKM)

Boxplot figure showing the RNA-seq estimated abundance levels for each annotated gene. The y-axis shows the FPKM values for each feature, the x-axes indicates the library. The y-axis is limited to 300 FPKM, removing the outliers (data outside this range represent genes with high changes in expression) to allow the visualization of the majority of genes. Boxes colored in yellow indicate libraries generated from cells grown in BMW media, red for YPD media and hypoxic conditions, green for YPD media and normoxic conditions, blue for the *upc2* deletion library, orange for cells grown at 37ºC. Each box contains 50% of the distribution centered on the median (black line). The first box in the BMW Library (yellow), and the first box in the YPD library in normoxic condition (green) represent libraries generated using a different protocol (78 bp read length) and were not used in expression analysis.

**Correlation values between biological replicates for wildtype cells grown in YPD in hypoxic conditions at 30ºC.**

|  | Rep1 | Rep2 | Rep3 | Rep4 |
| --- | --- | --- | --- | --- |
| Rep1 | 1 | 0.88 | 0.82 | 0.83 |
| Rep2 | 0.87 | 1 | 0.961 | 0.97 |
| Rep3 | 0.82 | 0.96 | 1 | 0.98 |
| Rep4 | 0.83 | 0.97 | 0.98 | 1 |

range: 0.96 >r> 0.82

**Correlation values between biological replicates for *upc2* deletion grown in YPD in hypoxic conditions at 30ºC.**

|  | Rep1* | Rep2 |
| --- | --- | --- |
| Rep1 | 1 | 0.96 |
| Rep2 | 0.96 | 1 |

**Correlation values between biological replicates for wildtype cells grown in BMW in normoxic conditions at 30ºC.**

|  | Rep1 | Rep2 | Rep3 | Rep4 | Rep5 | Rep6 |
| --- | --- | --- | --- | --- | --- | --- |
| Rep1 | 1.00 | 0.95 | 0.99 | 1.00 | 0.95 | 0.98 |
| Rep2 | 0.95 | 1.00 | 0.99 | 0.95 | 1.00 | 0.99 |
| Rep3 | 0.99 | 0.99 | 1.00 | 0.99 | 0.98 | 1.00 |
| Rep4 | 1.00 | 0.95 | 0.99 | 1.00 | 0.95 | 0.98 |
| Rep5 | 0.95 | 1.00 | 0.98 | 0.95 | 1.00 | 0.99 |
| Rep6 | 0.98 | 0.99 | 1.00 | 0.98 | 0.99 | 1.00 |

1 >r> 0.95 (the long-read library is not included in this comparison).

**Correlation values between biological replicates for wildtype cells grown in YPD in normoxic conditions at 30ºC.**

|  | Rep1 | Rep2 | Rep3 | Rep4 | Rep5 | Rep6 | Rep7 | Rep8 | Rep9 |
| --- | --- | --- | --- | --- | --- | --- | --- | --- | --- |
| Rep1 | 1.00 | 0.95 | 0.95 | 0.98 | 1.00 | 0.94 | 0.93 | 0.97 | 0.94 |
| Rep2 | 0.95 | 1.00 | 1.00 | 0.99 | 0.96 | 1.00 | 1.00 | 0.99 | 0.93 |
| Rep3 | 0.95 | 1.00 | 1.00 | 0.99 | 0.96 | 1.00 | 1.00 | 0.99 | 0.93 |
| Rep4 | 0.98 | 0.99 | 0.99 | 1.00 | 0.99 | 0.99 | 0.98 | 1.00 | 0.94 |
| Rep5 | 1.00 | 0.96 | 0.96 | 0.99 | 1.00 | 0.96 | 0.95 | 0.98 | 0.94 |
| Rep6 | 0.94 | 1.00 | 1.00 | 0.99 | 0.96 | 1.00 | 1.00 | 0.99 | 0.93 |
| Rep7 | 0.93 | 1.00 | 1.00 | 0.98 | 0.95 | 1.00 | 1.00 | 0.99 | 0.92 |
| Rep8 | 0.97 | 0.99 | 0.99 | 1.00 | 0.98 | 0.99 | 0.99 | 1.00 | 0.94 |
| Rep9 | 0.94 | 0.93 | 0.93 | 0.94 | 0.94 | 0.93 | 0.92 | 0.94 | 1.00 |

range: 1>r>0.92 (the long-read library is not included in this comparison).
