# Supplementary material for: Using RNA-seq to determine the transcriptional landscape and the hypoxic response of the pathogenic yeast Candida parapsilosis
Source: BMC Genomics. 2011 Dec 22;12:628. doi: 10.1186/1471-2164-12-628 (PMC3287387; doi:10.1186/1471-2164-12-628)
Supplement: Additional file 16 — Strains used in this study. List of the strains used in this study. [file 1471-2164-12-628-S16.DOC]

# Additional file 16: Strains used in this study.

| **Strains** | **Genotype** | **Description** |
| --- | --- | --- |
| *C. parapsilosis* CLIB214 | Wild type | Type strain |
| C. parapsilosis CDUhis1 | *ura3Δ::FRT/ ura3Δ::FRT*  *his1Δ::SAT1-FLP/ HIS1* | This study, derived from CDU1 |
| *C. parapsilosis* CDUhis11 | *ura3Δ::FRT/ ura3Δ::FRT*  *his1D::FRT/ HIS1* | This study, derived from CDUhis1 |
| *C. parapsilosis* CDUH1 | *ura3Δ::FRT/ ura3Δ::FRT*  *his1Δ::FRT/ his1Δ::SAT1-FLP* | This study, derived from CDUhis11 |
| *C. parapsilosis* CDUH3 | *ura3Δ::FRT/ ura3Δ::FRT*  *his1Δ::FRT/ his1Δ::FRT* | This study, derived from CDUH1 |
| *C. parapsilosis* CDupc5 | *upc2::HIS1*/*upc2::URA3*; *ura3/ura3, his1/his1* | This study, derived from CDUH1 |
| *C. parapsilosis* CDupc7 | *upc2Δ::FRT/upc2Δ::FRT* | Both *upc2* alleles deleted using *SAT1* flipper (unpublished) |
| *C. albicans* SC5314 | Wild type |  |
| *C. albicans* TW14920 | *upc2::URA3/upc2::ARG4* |  |

1. Ding C, Butler G: **Development of a gene knockout system in *Candida parapsilosis* reveals a conserved role for *BCR1* in biofilm formation**. *Eukaryot Cell* 2007, **6**:1310-1319.

2. Gillum AM, Tsay EY, Kirsch DR: **Isolation of the *Candida albican*s gene for orotidine-5'-phosphate decarboxylase by complementation of *S. cerevisiae ura3* and *E. coli pyrF* mutations**. *Mol Gen Genet* 1984, **198**(1):179-182.

3. Silver PM, Oliver BG, White TC: **Role of *Candida albicans* transcription factor Upc2p in drug resistance and sterol metabolism**. *Eukaryot Cell* 2004, **3**(6):1391-1397.
