# Supplementary material for: Using RNA-seq to determine the transcriptional landscape and the hypoxic response of the pathogenic yeast Candida parapsilosis
Source: BMC Genomics. 2011 Dec 22;12:628. doi: 10.1186/1471-2164-12-628 (PMC3287387; doi:10.1186/1471-2164-12-628)
Supplement: Additional file 17 — Oligonucleotide primers. List of oligonucleotide primers used in RT-PCR and to validate constructs and introns. [file 1471-2164-12-628-S17.DOC]

# Additional file 17: Oligonucleotide primers.

| **Oligo Names** | **Application** | **Sequence** |
| --- | --- | --- |
| upcuh_f | Knockout | ATGCTACTGAAGGATCTCGAAGATGCCTTACA  AGGATATCAAACGAGGAAAGAAGCGCCAGAG  AGCACGAGAAGAGTTTCGGTAACGCCAGGGT  TTTCCC |
| upcuh_r | Knockout | GTTGATAAACCCTTGATCTCCTTGCATTATATC  CAACTCTCTTGTAATATCACTCGGTAAATTATT  AGTATTGGTCATTTAAACAGCTATGACCATGA  TT |
| upcup | Screening | TTTCTATACGACGAGTGATACT |
| upcmid | Screening | GATACCGCCTTCGCCTTCTTCAG |
| URAR | Screening | TATAAACCAGTGTGTATGGGGTTG |
| HISR | Screening | TCGGTAGTTGGTGGTTAAGTAAAAG |
| 205890_F | intron validation | CCCATAAGCAGAAAAATTTGGA |
| 205890_R | intron validation | AAACGTTTTCGTGTGCCATA |
| 209350_F | intron validation | cgttagtgcaatatttatacaagca |
| 209350_R | intron validation | TCGATTGTCCAATACCAAAGTT |
| 400980_F | intron validation | caaaggtatattcaacgataacga |
| 400980_R | intron validation | CACCAGAGGCTTTCTTGACC |
| 405840_F | intron validation | tcgatttggcaccagaattt |
| 405840_R | intron validation | aacttctctccgtccgaggt |
| 601470_F | intron validation | Cctgcacaattgatcaagca |
| 601470_1R | intron validation | Aaccctatttgagaaaaactgaaa |
| 601470_2R | intron validation | tgaaataaccttgtcaattgaatataa |
| 603230_F | intron validation | Attgtgcaccacaagggaga |
| 603230_R | intron validation | acaccggatcgtttcaagtc |
| 807560_F | intron validation | cagcatagaacatggttgacg |
| 807560_R | intron validation | tgacaacagctggtctttcg |
| 601830_F | intron validation | Caggatcaggagcttcagga |
| 601830_R | intron validation | Gcagaacccttcatttcacc |
| 803400_F | intron validation | tgtttgaaaggggatctagaaga |
| 803400_R | intron validation | ctgattgaccacctggacct |
